# An Adhesive Hydrogel Technology for Enhanced Cartilage Repair: A Preliminary Proof of Concept

**DOI:** 10.3390/gels10100657

**Published:** 2024-10-14

**Authors:** Peyman Karami, Robin Martin, Alexis Laurent, Hui Yin Nam, Virginie Philippe, Lee Ann Applegate, Dominique P. Pioletti

**Affiliations:** 1 Department of Orthopedic Surgery and Traumatology, Lausanne University Hospital, University of Lausanne, CH-1011 Lausanne, Switzerland; peyman.karami@epfl.ch (P.K.); robin.martin@chuv.ch (R.M.); virginie.philippe@chuv.ch (V.P.); 2 Laboratory of Biomechanical Orthopaedics, Institute of Bioengineering, School of Engineering, EPFL, CH-1015 Lausanne, Switzerland; 3 Manufacturing Department, LAM Biotechnologies SA, CH-1066 Epalinges, Switzerland; alexis.laurent@unil.ch; 4 Regenerative Therapy Unit, Reconstructive and Hand Surgery Service, Lausanne University Hospital, University of Lausanne, CH-1066 Epalinges, Switzerland; lee.laurent-applegate@chuv.ch; 5 Department of Orthopaedic Surgery (NOCERAL), Faculty of Medicine, Universiti Malaya, Kuala Lumpur 50603, Malaysia; huiyin26@yahoo.com; 6 Center for Applied Biotechnology and Molecular Medicine, University of Zurich, CH-8057 Zurich, Switzerland; 7 Oxford OSCAR Suzhou Center, Oxford University, Suzhou 215123, China

**Keywords:** hydrogel, adhesion, cartilage, lateral integration

## Abstract

Knee cartilage has limited natural healing capacity, complicating the development of effective treatment plans. Current non-cell-based therapies (e.g., microfracture) result in poor repair cartilage mechanical properties, low durability, and suboptimal tissue integration. Advanced treatments, such as autologous chondrocyte implantation, face challenges including cell leakage and inhomogeneous distribution. Successful cell therapy relies on prolonged retention of therapeutic biologicals at the implantation site, yet the optimal integration of implanted material into the surrounding healthy tissue remains an unmet need. This study evaluated the effectiveness of a newly developed photo-curable adhesive hydrogel for cartilage repair, focusing on adhesion properties, integration performance, and ability to support tissue regeneration. The proposed hydrogel design exhibited significant adhesion strength, outperforming commercial adhesives such as fibrin-based glues. An in vivo goat model was used to evaluate the hydrogels’ adhesion properties and long-term integration into full-thickness cartilage defects over six months. Results showed that cell-free hydrogel-treated defects achieved superior integration with surrounding tissue and enhanced cartilage repair, with notable lateral integration. In vitro results further demonstrated high cell viability, robust matrix production, and successful cell encapsulation within the hydrogel matrix. These findings highlight the potential of adhesive hydrogel formulations to improve the efficacy of cell-based therapies, offering a potentially superior treatment for knee cartilage defects.

## 1. Introduction

Technical and therapeutic deficiencies in treating knee cartilage lesions pose a challenge in current medical practice. Available treatment methods vary depending on the lesion’s size and severity. Procedures for repairing cartilage aim to regenerate, restore, or replace articular cartilage to alleviate joint pain and hinder osteoarthritis progression. Despite a notable increase in cartilage repair procedures, the inherent absence of vascularity and poor cartilage tissue regeneration complicates effective treatment [1,2].

Common treatments for chondral injuries (i.e., typically under 2 cm^2^ in size) include osteochondral autograft transfer (mosaicplasty) or microfracture techniques. However, they may result in mechanically inferior cartilage that might necessitate additional interventions. Larger defects are often treated with cell-based therapies like different generations of autologous chondrocyte implantation (ACI). Despite improved long-term healing effects in advanced ACI treatments, challenges such as leakage of the injected cell suspension between membrane sutures, cell distribution in the defect site, and variable outcomes are encountered [3,4]. Moreover, lateral integration at the interface with the surrounding healthy cartilage tissue is a major challenge within the available therapies [5,6].

Adhesive hydrogels have gained attention as innovative solutions in cartilage tissue engineering [7,8,9]. Appropriate adhesive properties allow us to securely attach the hydrogel to chondral defect sites, promoting cellular infiltration and integration with adjacent tissues. Additionally, hydrogel scaffolds could mimic the native extracellular matrix (ECM) environment, creating an optimal setting for cell growth, differentiation, and cartilage regeneration [10]. However, current hydrogel systems face issues with the integration of materials used to fill cartilage tissue defects, mainly due to their poor adhesion properties. Liquid tissue adhesives are known for their slow bonding processes and weak bond strength. While notable advances have been made in hydrogel systems such as pre-formed patches and dry tapes, the challenge remains in developing injectable fast-curing hydrogels with robust adhesion attributes, capable of withstanding knee mechanical loads [11,12]. Furthermore, commercial bioadhesive glues, such as fibrin-based glues, exhibit weak mechanical and adhesive performance [13]. Therefore, developing hydrogels that combine intrinsic adhesion with optimal injectability for repairing cartilage is a key milestone yet to be achieved.

Recently, we developed a family of injectable photocurable adhesive hydrogels with a wide range of tunable physicochemical properties [12]. In this study, we present a proof-of-concept for our hydrogel formulations as a promising adhesive scaffold platform for the repair of articular cartilage lesions. We demonstrated successful lateral integration outcomes through an in vivo study on a goat model using a cell-free hydrogel device. Furthermore, we have conducted an in vitro study by encapsulating bovine chondrocytes in the hydrogel to evaluate potential cell delivery to enhance pro-regeneration outcomes. Our research shows the potential of our hydrogel formulation to improve the effectiveness of chondrocyte-based therapies for knee cartilage defects, providing an efficient and potentially superior treatment strategy.

## 2. Results and Discussion

**Figure 1:**
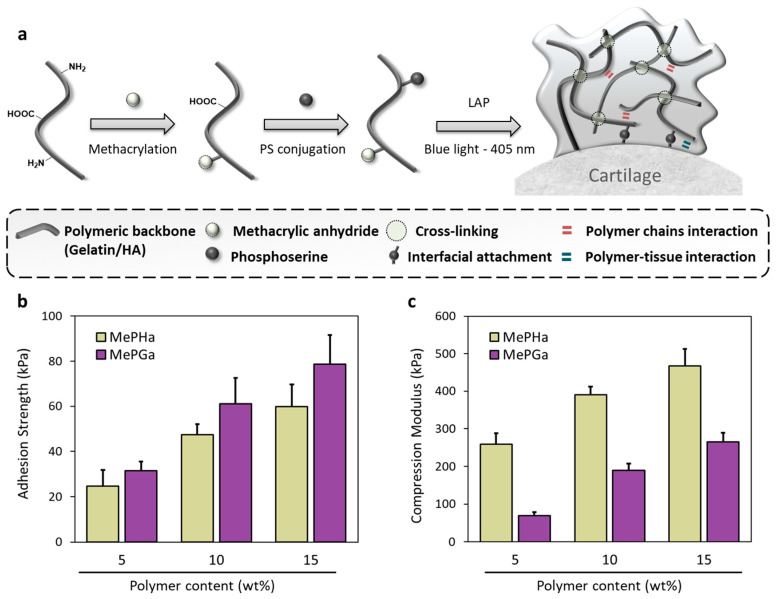
(**a**) Schematic illustration of the synthesis process for MePHa and MePGa hydrogels. (**b**) The tensile adhesion strength and (**c**) compressive modulus at different polymer content for MePHa and MePGa hydrogels. (*n* = 3).

Figure 1a illustrates the schematic fabrication process of the adhesive hydrogel formulation designed for cartilage repair. The process involved methacrylation of the polymeric backbone (gelatin/HA) followed by conjugation with phosphoserine (PS). Subsequent cross-linking using LAP (Lithium phenyl-2,4,6-trimethylbenzoylphosphinate) under blue light (405 nm) resulted in the hydrogel formation. This synthesis aims to enhance the adhesive properties of the hydrogel to cartilage by promoting interfacial attachment to the host tissue. The comprehensive characterization of our hydrogel system, including adhesion mechanism, physicochemical properties, mechanical performance, and degradation profiles, has been previously presented [12]. This prior work provides a solid foundation for understanding the performance attributes and potential applications of the hydrogels.

The tensile adhesion strength of the developed MePHa and MePGa hydrogels was measured for different polymer contents (5, 10, and 15 wt%). Results indicated that MePHa and MePGa hydrogels exhibit increasing adhesion strength with higher polymer contents (Figure 1b). Even with a high water content of 95 wt%, both hydrogels achieve adhesion strengths higher than that of fibrin glue (~14 kPa), which is a widely used adhesive specifically for providing sealing in ACI methods or to secure cells in place within the defect site. In addition to weak adhesion strength, fibrin glue has poor mechanical properties to withstand mechanical stresses and degrades quickly in vivo [14]. This can compromise the structural integrity and durability attributes, leading to premature loss of the adhesive function and migration of the implanted material before the tissue has sufficiently healed.

MePHa and MePGa hydrogels (Figure 1c) exhibit increasing compressive modulus with higher polymer content. At 15% polymer content, MePHa achieves a compressive modulus of around 468.2 ± 44.2 kPa, and MePGa around 264.9 ± 25.6 kPa. These values indicate significantly better mechanical performance than fibrin glue, which typically does not offer substantial mechanical support.

To further substantiate our findings and assess the long-term performance and biocompatibility of the hydrogels, we conducted a proof-of-concept in vivo evaluation of the adhesive performance of the hydrogel systems and investigated the biological interactions at the molecular level in vitro.

**Figure 2:**
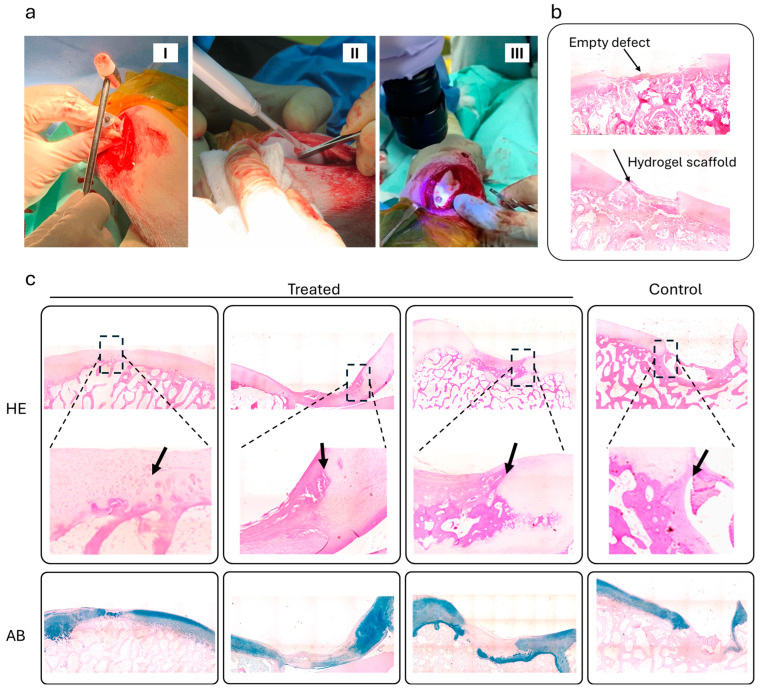
Repair effects of the bioadhesive hydrogel in full-thickness cartilage defects. (**a**) In vivo implantation procedure of the bioadhesive scaffold in a large animal model: (**I**) creation of a full-thickness cartilage defect in the medial femoral groove in the knee joint; (**II**) filling the defect by injecting bioadhesive precursor; and (**III**) immediate photo-curing in situ. (**b**) H&E staining images of the defect sites after 2 days of implantation. (**c**) H&E and AB staining images from the treated versus control defects after 6 months of implantation. The control samples represent the cartilage defect treated without bioadhesive hydrogel injection.

The in vivo study was conducted on a goat model to assess the adhesive performance of the developed hydrogel systems. The surgical procedure involved creating a cartilage defect and applying the hydrogel, followed by photo-curing with blue light to ensure proper adhesion (Figure 2a). Figure 2b shows the histological comparison between an empty control defect and the treated defect with the hydrogel scaffold after 2 days of implantation. Unlike the control defect, the hydrogel-treated defect demonstrated initial signs of tissue integration to the surrounding cartilage. This highlights the hydrogel’s capability to stay in the implanted site and promote early-stage tissue repair. Besides adhesive contact, the hydrogel may facilitate ECM deposition and cellular migration at the interface, potentially supporting matrix organization. Over time, this biological activity, along with mechanical matching and the hydrogel’s scaffold-like properties, could contribute to the stability and long-term integration of the hydrogel within the native tissue.

Figure 2c provides the long-term histological analysis of treated versus control samples after 6 months of implantation. The H&E staining images for the treated samples indicate successful lateral integration of the hydrogel scaffold into the surrounding cartilage tissue (arrows), while control samples without hydrogel show poor integration and significant gaps at the interface defect site. This is also evidenced by the rarely observed vertical fissures at the repair interface, in contrast to the control group, which displayed notable fissures. Alcian blue (AB) staining further supports these findings, with treated samples exhibiting enhanced cartilage repair and matrix production compared to controls and a perfect alignment at the interface. However, the non-intense staining, indicative of low glycosaminoglycan (GAG) content, suggests that the new tissue formation is mainly fibrocartilage, as no cells or biological drugs were used with the hydrogel in the present study. No adverse immune or inflammatory reactions were observed, supporting the hydrogel biocompatibility within the joint space.

Therefore, this engineered bioadhesive physicochemical performance provides a good basis for this formulation also being used as injectable cell-laden scaffolds for tissue engineering and drug delivery applications. To further demonstrate this capacity, we conducted an in vitro experiment to study the differentiation capacity of bovine chondrocytes encapsulated in the adhesive MePHa-gel and MePGa-gel. The study demonstrated that both adhesive hydrogels supported high cell viability, proliferation, and matrix production, with the gelatin-based MePGa-gel showing superior chondro-inductive properties and mechanical performance. Significant upregulation of chondrogenic markers and downregulation of the catabolic marker ADAMTS5 further highlighted the potential of these hydrogels for cartilage regeneration (See the Supplementary Information and Figure S1 for details).

## 3. Conclusions

The in vivo results with MePGa-gel demonstrate that the proposed photocurable adhesive hydrogel system offers a promising solution for treating articular cartilage lesions. The hydrogels exhibited robust adhesion to the defect site and promoted tissue integration, which is critical for effective regeneration. Furthermore, the characterization of the hydrogel system demonstrates its ability to provide a proper microenvironment to facilitate an optimal setting for cell growth, differentiation, and tissue regeneration. The successful lateral integration observed in the goat model addresses a major challenge in current cartilage repair techniques as there are frequent problems at the interface. This suggests the potential of our hydrogel system to improve the outcomes of cell-based therapies for knee cartilage defects.

Namely, our promising outcome has significant potential for clinical application in cartilage repair through the following unique advantages. It provides strong integration between original and repaired cartilage and helps tissue restore its function. In advanced ACI therapies, the adhesive hydrogel approach can enhance surgical procedures by eliminating the need for membranes for cell retention and addressing the issue of cell leakage and cell distribution. Moreover, through improved cell retention, the hydrogel adhesiveness ensures that therapeutic cells remain anchored within the damaged cartilage area and facilitates prolonged exertion of therapeutic mechanisms of action, including extracellular matrix synthesis and overall cartilage healing. Therefore, in a wider perspective, a therapeutic protocol using adhesive hydrogel devices for optimized therapeutic delivery can enhance tissue repair and function and positively impact patient quality of life and mobility. Our future studies will focus on establishing a cell-based therapeutic protocol using human cells through comprehensive in vitro and in vivo investigations to further validate the hydrogel’s efficacy and safety attributes.

## 4. Materials and Methods

Gelatin Type A, derived from porcine skin (ref. G2500), methacrylic anhydride (ref. 276685), sodium hydroxide (ref. 71690), and hydrochloric acid were obtained from Sigma Aldrich (Saint Louis, MO, USA). Sodium hyaluronate (HA) was procured from Contipro (ref. 00-52-18, Dolní Dobrouč, Czech Republic). Phosphoserine (ref. 17885-08-4) was sourced from Flamma (Bergamo, Italy). EDC (N-(3-Dimethylaminopropyl)-N′-ethylcarbodiimide hydrochloride, ref. 03450) was supplied by Sigma Aldrich, and Sulfo-NHS (N-hydroxysulfosuccinimide, ref. 24510) was provided by Thermo Fisher Scientific (Rockford, IL, USA). LAP (Lithium Phenyl(2,4,6-trimethylbenzoyl)phosphinate, ref. 6146) was purchased from Tocris Bioscience (Bristol, UK) for radical polymerization.

### 4.1. Synthesis of Pre-Polymers

In this study, we used two hydrogel formulations, MePHa (methacrylated phosphoserine-containing hyaluronic acid) and MePGa (methacrylated phosphoserine-containing gelatin), previously introduced in our earlier work, where we presented a family of injectable adhesive hydrogels based on a novel design paradigm for adhesive hydrogel networks [12]. This innovative approach addresses the challenge of combining proper injectability with strong intrinsic adhesion in hydrogels by achieving synergy between interfacial chemistry and bulk mechanical properties. Hydrogel pre-polymers were developed through a two-step process that enhances their adhesive properties, a crucial feature for successful cartilage repair.

The synthesis of MePGa involved the methacrylation of gelatin followed by conjugation with phosphoserine. Accordingly, 5 g of gelatin type A were dissolved in 50 mL of Dulbecco’s PBS (DPBS) at 60 °C for 30 min. Methacrylic anhydride (2 mL) was added gradually at a rate of 0.5 mL/min, and the reaction mixture was stirred at 50 °C for 2 h. The reaction was halted by diluting the mixture with DPBS four times. The solution underwent dialysis against distilled water at 50 °C using a 12–14 kDa dialysis membrane for 5 days, followed by lyophilization for 4 days. In the second step, 0.5 g of methacrylated gelatin was dissolved in 25 mL of MES buffer at 50 °C, adjusting the pH to 5. Following EDC/NHS activation of carboxyl groups at 37 °C, phosphoserine (25 mM) was introduced, and the mixture was stirred continuously to form amide bonding. The resultant mixture was filtered, dialyzed against distilled water for 5 days, and lyophilized for 4 days to obtain a solid porous product which was stored at 4 °C.

The synthesis of MePHa involved a two-step procedure. In brief, 1 g of hyaluronic acid was dissolved in 50 mL of deionized water inside a glass container and stirred vigorously for 30 min at ambient temperature. The container was then cooled in an ice bath to maintain the temperature at 4 °C. While stirring, the pH of the HA solution was adjusted to 8.5 using 1.0 M NaOH. Then, 1.5 mL methacrylic anhydride were slowly introduced into the mixture, keeping the pH at around 8 ± 0.5. Following three h of maintaining pH levels, the solution was stirred overnight at 4 °C in a flask covered with parafilm. The mixture was then placed into 50 mL tubes and spun in a centrifuge for 5 min at 1200× *g*. The solution was dialyzed using a membrane with a weight cutoff of 6–8 kDa for three days, with the water being changed twice daily. Following dialysis, the solution was frozen and freeze-dried for 3 days. For phosphoserine conjugation, 1 g of the freeze-dried polymer was dissolved in 50 mL of buffer, and the pH was adjusted to 5. The carboxyl groups on the polymer chain were activated using EDC while being stirred continuously for 15 min. Subsequently, phosphoserine (25 mM) was added, and the reaction was allowed to continue for six h. The resulting mixture was filtered, dialyzed, and freeze-dried for 3 days.

Hydrogel fabrication. Hydrogel precursors were prepared by dissolving the lyophilized polymers and LAP photoinitiator (0.02% *w*/*v*) in PBS. After homogenizing the precursors, they were cross-linked by exposure to 405 nm light.

### 4.2. In Vivo Study

#### 4.2.1. Animal Model and Surgical Procedures

Animal surgeries were conducted on three 2-year-old adult male Boer goats. The approval for the in vivo study was granted by the Universiti Malaya Institutional Animal Care and Use Committee (UM IACUC) at the Universiti Malaya (Kuala Lumpur, Malaysia). Three goats, each weighing around 33 kg, were included in the experiments. The animals underwent health assessments, including blood and urine analyses, to confirm their well-being. Before surgery, a fasting period of 18 h was administered although the animals had access to water.

General anesthesia was induced through injections of ketamine (3–5 mg/kg) and diazepam (0.2 mg/kg). Preemptive pain relief was administered via a dose of meloxicam (0.2 mg/kg). The surgeries were carried out under conditions with the goats intubated and connected to an oxygen supply throughout the procedure, maintaining anesthesia with isoflurane. Ophthalmic ointment was used to safeguard their corneas during the surgery. A medial parapatellar incision was made to reach the left knee joint. Full-thickness cartilage defects measuring 5 mm in diameter each were created in the trochlear groove of the femur in the left knee using a chondrotome. Subsequently, the defect was filled with sterilized MePGa-gel precursor at 15 wt% of polymer content and promptly photocured in place using a portable light source, emitting at a wavelength of 405 nm. The light was applied directly to the filled defects for 1 min to ensure complete curing of the gel. For comparison, control defects were not treated. No immobilization was applied after the surgery and the animals were in full weight-bearing condition. Pain relief after the operation involved administering injections of meloxicam (0.1 mg/kg) once a day for two days. The animals were euthanized at two time points, one animal at two days and three animals at six months after implantation, for a thorough examination. Additionally, for each time point, one animal was considered as the control.

#### 4.2.2. Histological Analysis

After euthanasia, the knee joints were collected to assess the repair and integrity of the implanted hydrogel at defect sites. The specimens were fixed overnight using 10% buffered formalin then decalcified with formic acid, dehydrated using graded ethanol solutions from 70% to 100%, treated with xylene, and embedded in paraffin for sectioning. The histological sections were cut from the middle of the defect. The samples were stained with hematoxylin, eosin (H&E), and alcian blue to evaluate matrix architecture and glycosaminoglycan content.

### 4.3. Primary Cell Culture

Primary chondrocytes from bovine knee joints were isolated and cultured in standard T75 flasks at 37 °C. Cells were expanded in DMEM, supplemented with 10% Fetal Bovine Serum (FBS), 1% Penicillin–Streptomycin (PS), and 1% L-Glutamine. The cultured chondrocytes were suspended in hydrogel precursors at a polymer concentration of 5.5%, with a cell density of 10 million cells per milliliter. The precursors were photocrosslinked in cylindrical molds (5 mm in diameter, 2.2 mm in height) using 405 nm light at an intensity of 10 mW/cm^2^. The encapsulated constructs were then cultured in a freshly prepared chondrogenic medium, containing 50 µg/mL vitamin C, 10 ng/mL TGF-β3, and diluted ITS IV, in serum-free DMEM supplemented with 1% PS and 1% L-Glutamine. The culture medium was refreshed every 3 days for a period of up to four weeks. Cell behavior, extracellular matrix formation, and microtissue development were assessed during this time.

#### 4.3.1. Cell Viability and Distribution Analysis

Cell viability in the constructs was assessed using the Live-Dead Assay Kit (Biotium, Fremont, CA, USA), following the manufacturer’s protocol. A staining solution comprising calcein AM and ethidium homodimer was prepared by adding the dyes to PBS at concentrations of 0.2 μL/mL and 0.4 μL/mL, respectively. The constructs were cut into halves and incubated in the staining solution for 20 min. After staining, the samples were rinsed with PBS and visualized under a Zeiss LSM 700 confocal microscope (Carl Zeiss, Jena, Germany) at a magnification of 20×. Fluorescence images were captured using appropriate filters for green and red dyes. In addition to the live/dead analysis, a custom FIJI script was used to quantify cell viability from z-stack images acquired through confocal microscopy.

#### 4.3.2. Biochemical Analysis

To evaluate DNA content and the production of sulfated glycosaminoglycans (sGAG) in the constructs, biochemical assays were performed at various time points. At each time point, samples were enzymatically digested and transferred to 1.5 mL Eppendorf tubes, where 500 µL of papain digestion buffer containing 6 µL/mL papain (Sigma P-3125) was added. The papain solution was prepared by dissolving 7.1 g sodium phosphate dibasic (Na_2_HPO_4_), 1.86 g EDTA, and 870 mg L-cysteine HCl in 500 mL deionized water, with the pH adjusted to 6.5. After crushing and mincing of the samples, they were incubated overnight at 60 °C to ensure full digestion. sGAG quantification was conducted using the 1,9-dimethylmethylene blue (DMMB) assay with shark chondroitin sulfate (CS). For each sample, 20 µL of the digest was loaded into a 96-well plate, followed by 130 µL of DMMB dye in triplicate. Absorbance at 590 nm was measured using a Wallac 1420 Victor2 microplate reader (PerkinElmer, Ramsey, MN, USA). DNA content was analyzed using the Hoechst 33,258 fluorescence assay (Thermo Fisher Scientific) by adding 10 µL of sample to 140 µL of Hoechst dye in a black 96-well plate. The emission signal at 460 nm was recorded, and the DNA concentration was calculated using a standard curve generated with Calf Thymus DNA (Thermo Fisher Scientific, Waltham, MA, USA).

#### 4.3.3. Gene Expression Analysis

RNA extraction for chondrogenic gene expression analysis was performed at each time point using real-time PCR. Hydrogel constructs were transferred to Eppendorf tubes, and 300 µL of Trizol reagent was added. Samples were homogenized on ice using a pestle, and RNA was isolated following the NucleoSpin RNA Kit (Macherey-Nagel, Düren, Nordrhein-Westfalen, Germany) protocol. RNA concentration was determined using a Nanodrop Lite Spectrophotometer (Thermo Fisher Scientific, Waltham, MA, USA). For reverse transcription, 1 µg of RNA was used to synthesize complementary DNA (cDNA) with TaqMan^®^ Reverse Transcription Reagents (Applied Biosystems, Waltham, MA, USA). Gene expression was quantified using SybrGreen^®^ Mastermix (Applied Biosystems) on a StepOnePlus™ Real-Time PCR System (Applied Biosystems, Waltham, USA). The expression of genes related to chondrogenesis, including COL2a1 (collagen type II), ACAN (aggrecan), and ADAMTS5, was measured. RPL13a was selected as the reference gene. PCR conditions included an initial denaturation at 95 °C for 1 min, followed by 40 cycles of 95 °C for 5 s and 60 °C for 30 s. Relative gene expression was determined using the ΔΔCT method.

## Data Availability

The original contributions presented in the study are included in the article/Supplementary Materials, further inquiries can be directed to the corresponding author.

## Supplementary Materials

The following supporting information can be downloaded at https://www.mdpi.com/article/10.3390/gels10100657/s1. Supplementary results for the in vitro cell-based experiments. Figure S1: Results of the in vitro cell-based experiments [15,16].

## Abbreviations

AB	Alcian blue
ACI	autologous chondrocyte implantation
cDNA	complementary DNA
CS	chondroitin sulfate
DMEM	Dulbecco’s modified Eagle medium
DMMB	1,9-dimethylmethylene blue
DNA	deoxyribonucleic acid
DPBS	Dulbecco’s phosphate buffered saline
ECM	extracellular matrix
EDTA	ethylenediaminetetraacetic acid
FBS	fetal bovine serum
GAG	glycosaminoglycan
HA	hyaluronic acid
H&E	haematoxylin and eosin
LAP	lithium phenyl-2,4,6-trimethylbenzoylphosphinate
PBS	phosphate-buffered saline
PCR	polymerase chain reaction
PS	penicillin-streptomycin
PS	phosphoserine
RNA	ribonucleic acid
