# Supplementary material for: An Adhesive Hydrogel Technology for Enhanced Cartilage Repair: A Preliminary Proof of Concept
Source: Gels. 2024 Oct 14;10(10):657. doi: 10.3390/gels10100657 (PMC11507104; doi:10.3390/gels10100657)
Supplement: Supplementary file 1 [file gels-10-00657-s001.zip › gels-3233492-supplementary.pdf]

Supplementary Information

An Adhesive Hydrogel Technology for Enhanced Cartilage Repair: A Preliminary Proof of Concept

Peyman Karami [1,2 †], Robin Martin [1 †], Alexis Laurent [3,4], Hui Yin Nam [5], Virginie Philippe [1,4], Lee Ann Applegate [4,6,7], Dominique P. Pioletti [2*]

[1] Department of Orthopedic Surgery and Traumatology, Lausanne University Hospital, University of Lausanne, CH-1011 Lausanne, Switzerland

[2] Laboratory of Biomechanical Orthopaedics, Institute of Bioengineering, School of Engineering, EPFL, CH-1015 Lausanne, Switzerland

[3] Manufacturing Department, LAM Biotechnologies SA, CH-1066 Epalinges, Switzerland

[4] Regenerative Therapy Unit, Reconstructive and Hand Surgery Service, Lausanne University Hospital, University of Lausanne, CH-1066 Epalinges, Switzerland

[5] Department of Orthopaedic Surgery (NOCERAL), Faculty of Medicine, Universiti Malaya, 50603 Kuala Lumpur, Malaysia

[6] Center for Applied Biotechnology and Molecular Medicine, University of Zurich, CH-8057 Zurich, Switzerland

[7] Oxford OSCAR Suzhou Center, Oxford University, Suzhou 215123, China

[†] Co-first authors

[*] Corresponding author, Email: dominique.pioletti@epfl.ch

In vitro analysis

The performed *in vitro* study evaluated the viability, proliferation, chondrogenic differentiation, and matrix production of the encapsulated cells, along with the physical characteristics of the 3D hydrogel constructs, over a 4-week period. Cells encapsulated within both adhesive hydrogels maintained a high level of viability (>84%) at all time points, with over 84% live cells after 4 weeks (Figure S1a and S1j). DNA content analysis revealed a significant increase, suggesting cell proliferation within the hydrogels (Figure S1b). Additionally, the accumulation of sulfated glycosaminoglycans (sGAG) increased significantly at 2 and 4 weeks of 3D culture (Figure S1c). Although the gelatin-based MePGa-Gel showed slightly lower cell viability and DNA content compared to the hyaluronic acid-based MePHa-Gel, it exhibited a greater capacity for sGAG deposition. This suggests that MePGa-Gel possesses superior chondro-inductive properties. Consequently, the mechanical properties of the cell-seeded hydrogels improved over time, with increases in both stiffness and energy dissipation capacity. However, the improvement in mechanical properties was more pronounced in the MePGa-Gel after 4 weeks, showing a stiffness of 1.51 ± 0.24 MPa and energy dissipation of 7338.52 ± 1653 kJ/m^3, which closely approximates the values found in native cartilage [1]. Gene expression analysis was conducted for key anabolic markers (Collagen type II and Aggrecan) and the catabolic marker ADAMTS5 using real-time RT-PCR. Both Collagen type II (COL2A1) and Aggrecan (ACAN) were significantly upregulated in the two types of hydrogels (p-value < 0.001) over the course of 4 weeks in 3D culture. In contrast, ADAMTS5 expression was greatly reduced in the hydrogel constructs, which is favorable for cartilage repair since ADAMTS5 is associated with the degradation of extracellular matrix components [2].

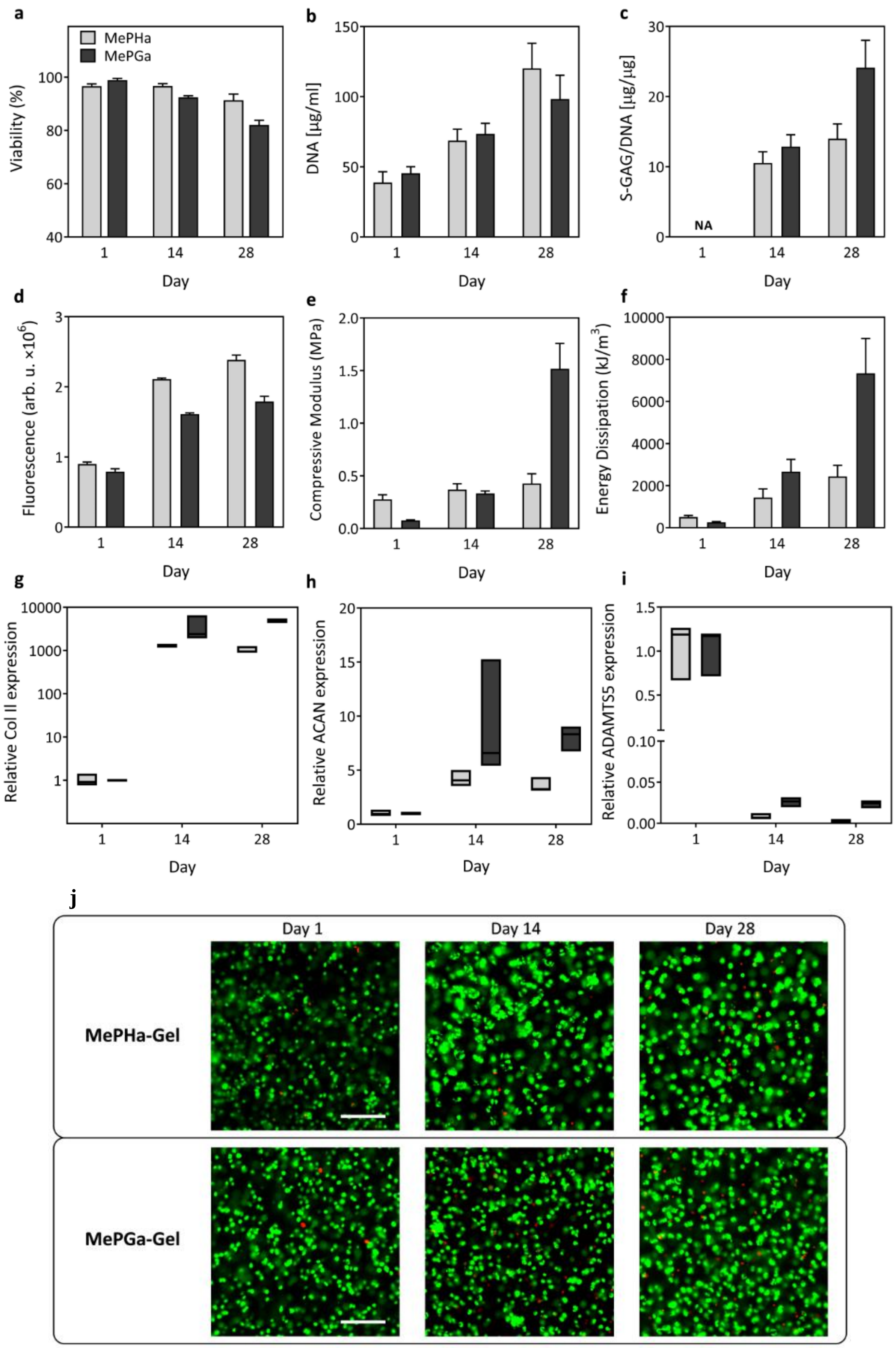
a
MePHa
MePGa
Viability (%)
Day
b
DNA [µg/ml]
Day
c
S-GAG/DNA [µg/µg]
NA
Day
d
Fluorescence (arb. u. ×10^6)
Day
e
Compressive Modulus (MPa)
Day
f
Energy Dissipation (kJ/m^3)
Day
g
Relative Col II expression
Day
h
Relative ACAN expression
Day
i
Relative ADAMTS5 expression
Day
j
Day 1
Day 14
Day 28
MePHa-Gel
MePGa-Gel

Figure S1. *In vitro* evaluation of the chondrocytes' biological response while encapsulated in adhesive hydrogels (WC: 95%) over 4 weeks of 3D culture. **(a)** Viability of chondrocytes in MePHa-Gel and MePGa-Gel constructs using a live/dead assay. **(b, c)** Biochemical analysis: DNA and sGAG/DNA content in hydrogel constructs. **(d)** Cell proliferation using a PrestoBlue™ assay. **(e, f)** Mechanical characterization. **(g-i)** Gene expression analysis: collagen type II (COL2A1), aggrecan (ACAN), and ADAMTS5. **(j)** Representative Live-Dead confocal images of encapsulated bovine chondrocytes at different time points after 3D encapsulation. Live cells are imaged using the green stain, and dead cells are shown using the red stain. Samples are cut into two halves, and images are taken from the cross-section. Results demonstrate that the cells are well distributed in the hydrogel constructs. Scale bars show 150 μm. (n = 3)

References

1. Mow, V.C.; Holmes, M.H.; Lai, W.M. Fluid transport and mechanical properties of articular cartilage: A review. *J. Biomechan.* **1984**, *17*, 377–394.
2. Rienks, M.; Barallobre-Barreiro, J.; Mayr, M. The emerging role of the ADAMTS family in vascular diseases. *Circul. Res.* **2018**, *123*, 1279–1281.
